# Implication of system x_c_^−^ in neuroinflammation during the onset and maintenance of neuropathic pain

**DOI:** 10.1186/s12974-024-03112-9

**Published:** 2024-05-07

**Authors:** Pauline Beckers, Inês Belo Do Nascimento, Mathilde Charlier, Nathalie Desmet, Ann Massie, Emmanuel Hermans

**Affiliations:** 1 Institute of Neuroscience, Group of Neuropharmacology, Université catholique de Louvain (UCLouvain), Avenue Hippocrate 53 (B1.53.01), Brussels, 1200 Belgium; 2 Neuro-Aging & Viro-Immunotherapy, Center for Neurosciences, Vrije Universiteit Brussel (VUB), Laarbeeklaan 103, Brussels, 1090 Belgium

**Keywords:** xCT, Slc7a11, Spinal cord, Central sensitization, Glutamate, Inflammation, Glial cells, Chronic pain

## Abstract

**Background:**

Despite the high prevalence of neuropathic pain, treating this neurological disease remains challenging, given the limited efficacy and numerous side effects associated with current therapies. The complexity in patient management is largely attributed to an incomplete understanding of the underlying pathological mechanisms. Central sensitization, that refers to the adaptation of the central nervous system to persistent inflammation and heightened excitatory transmission within pain pathways, stands as a significant contributor to persistent pain. Considering the role of the cystine/glutamate exchanger (also designated as system x_c_^−^) in modulating glutamate transmission and in supporting neuroinflammatory responses, we investigated the contribution of this exchanger in the development of neuropathic pain.

**Methods:**

We examined the implication of system x_c_^−^ by evaluating changes in the expression/activity of this exchanger in the dorsal spinal cord of mice after unilateral partial sciatic nerve ligation. In this surgical model of neuropathic pain, we also examined the consequence of the genetic suppression of system x_c_^−^ (using mice lacking the system x_c_^−^ specific subunit xCT) or its pharmacological manipulation (using the pharmacological inhibitor sulfasalazine) on the pain-associated behavioral responses. Finally, we assessed the glial activation and the inflammatory response in the spinal cord by measuring mRNA and protein levels of GFAP and selected M1 and M2 microglial markers.

**Results:**

The sciatic nerve lesion was found to upregulate system x_c_^−^ at the spinal level. The genetic deletion of xCT attenuated both the amplitude and the duration of the pain sensitization after nerve surgery, as evidenced by reduced responses to mechanical and thermal stimuli, and this was accompanied by reduced glial activation. Consistently, pharmacological inhibition of system x_c_^−^ had an analgesic effect in lesioned mice.

**Conclusion:**

Together, these observations provide evidence for a role of system x_c_^−^ in the biochemical processes underlying central sensitization. We propose that the reduced hypersensitivity observed in the transgenic mice lacking xCT or in sulfasalazine-treated mice is mediated by a reduced gliosis in the lumbar spinal cord and/or a shift in microglial M1/M2 polarization towards an anti-inflammatory phenotype in the absence of system x_c_^−^. These findings suggest that drugs targeting system x_c_^−^ could contribute to prevent or reduce neuropathic pain.

## Background

Neuropathic pain (NP) largely arises from a pathological imbalance between excitatory and inhibitory controls of pain transmission, especially at the level of the dorsal spinal cord [1–4]. Together with a persistent neuroinflammation, this imbalance promotes plastic adaptations in the central nervous system (CNS) that alter membrane excitability of neurons. This leads to an enhanced synaptic efficacy in pain pathways and is commonly referred as central sensitization [5]. Pro-inflammatory cytokines released by glial cells have been suggested to enhance neuronal and glial release of glutamate, the major excitatory neurotransmitter in the CNS [6, 7]. Neuroinflammation also promotes a transcriptional up-regulation of glutamate receptors, which leads to a persistent facilitatory influence on excitatory signals supporting pain transmission [8].

In different models of NP, the capacity of astrocytes to rapidly clear glutamate from the synaptic cleft was shown to be compromised [9–11]. Accordingly, pharmacological and genetic interventions aiming at increasing the expression or the functionality of high-affinity glutamate transporters (EAATs) have demonstrated promising analgesic effects in animal models of NP [12, 13]. To date, research on NP and the development of novel treatments targeting glutamate transmission have primarily focused on receptors and EAATs. As such, other molecular actors known to play an important role in the control of glutamate transmission, such as the cystine-glutamate exchanger (system x_c_^−^) have been overlooked.

Mainly expressed in glial cells, system x_c_^−^ is a transmembrane protein composed of two different subunits [14]. While the heavy chain, 4F2hc (SLC3A2), is shared among various plasma membrane solute carrier proteins, the light chain xCT (SLC7A11) determines substrate specificity and transport activity. System x_c_^−^ mediates the exchange of cystine for glutamate in a 1:1 molecular ratio, releasing glutamate into the extracellular space. Consequently, it provides cells with the cysteine, the rate-limiting amino acid residue for synthesizing antioxidant glutathione, but also indirectly reinforces glutamate signaling [15, 16]. Hence, system x_c_^−^ has even been identified as the major source of extracellular glutamate in several structures of the CNS [17, 18]. As for EAATs, a tight regulation of its expression and activity is essential to support protection against oxidative stress, but also to control extracellular glutamate concentration and neuronal excitation.

Considering its biochemical functions and its widespread distribution in the CNS, system x_c_^−^ has been studied in several neurological disorders in which dysregulated excitatory transmission contributes to nervous insults and neurodegeneration. In particular, increased xCT expression has been documented in models of Parkinson’s disease, amyotrophic lateral sclerosis, multiple sclerosis as well as in stroke or glioblastoma [19–24]. A role for system x_c_^−^ was also documented in the modulation of inflammatory responses, raising further interest for its implication in pathologies combining neuroinflammation and increased neuronal excitation [19]. Considering this, the present study aimed at examining a putative role for system x_c_^−^ in the modulation of nociceptive signals and associated neuroinflammation. To address this question, we have used a validated surgical mouse model of NP in which the expression and activity of system x_c_^−^ were manipulated by genetic and pharmacological approaches.

## Materials and methods

### Animals and ethical statement

All experiments were conducted in strict accordance with the recommendations of the European commission and with the agreement of the Belgian Ministry of Agriculture (code number LA 1,230,618). The ethical committee of the Université catholique de Louvain for animal experiments specifically approved this study (aggregation number 2019/UCL/MD/033). Wild-type (xCT^+/+^) and xCT knock-out (xCT^−/−^) mice used in this study were high-generation descendants (with C57BL/6J background) of the strain previously described [25]. These mice lacking xCT were generously provided by Pr. Hideyo Sato (Department of Medical Technology, Niigata University, Japan) and bred in the animal facilities of the Vrije Universiteit Brussel and the Université catholique de Louvain. All experiments combined homozygous animals obtained both from the breeding of heterozygous animals (F1) and from a second generation using homozygous parents (F2).

All animals were accommodated under standard laboratory conditions, receiving food and water ad libitum, and were housed in the animal facility at the Université catholique de Louvain, in a 12–12 h light-dark cycle, controlled temperature and humidity conditions. xCT knock-out mice were genotyped by end-point PCR on genomic DNA extracted from an ear biopsy using specific primers for the Slc7a11 gene. PCR amplification products were analyzed by agarose-gel electrophoresis to identify bands revealing the intact or truncated gene [25]. Since pain hypersensitivity in model of NP is sex-dependent, only female mice were herein used to reduce variability.

### Neuropathic pain model – partial sciatic nerve ligation (PSNL)

Adult female mice aged between 10 and 12 weeks were randomly subjected to PSNL or sham surgery using a procedure described earlier with minor modifications [26]. Briefly, mice were anesthetized with sevoflurane (4% for induction and 3% for maintenance with oxygen as a carrier gas). After isolation from the surrounding connective tissue, the left sciatic nerve was exposed. With an 8 − 0 suture, one half to one third of the nerve was tightly ligated above its trifurcation. Muscle and skin layers were finally closed with a 6 − 0 suture. Sham surgeries were performed by simply exposing the nerve before suturing the wound without performing the sciatic nerve ligation.

### Mechanical hypersensitivity – von frey hair filament test

Animals were placed in transparent chambers positioned on an elevated mesh floor. Acclimatization was allowed for 20 min after which the von Frey test was performed. Herein, a set of 10 calibrated von Frey hair filaments (Stoelting Co., Wood Dale, IL) was used (0.04, 0.07, 0.16, 0.4, 0.6, 1, 1.4, 2, 4 and 6 g). Filaments were applied perpendicular to the plantar surface of the hind paw starting with the 0.4 g filament.

To determine the mechanical sensitivity, the “up and down” method was used [27]. Briefly, the choice for the following filament was based on the previous filament response, being the closest-lower filament in case of a positive response or the closest-higher filament in case of a negative response. A positive response was defined by a paw withdrawal associated with aversive behavior, such as licking and/or shaking the stimulated paw. This method of von Frey filament application was continued until a sequence of nine filament applications was completed. The 50% paw withdrawal threshold (PWT) was then calculated using the formula previously described [28]. The experimenter responsible for conducting the behavioral tests was blind to the treatment, the genotype and the surgery performed on the mice. The cumulative changes in PWT from baseline values throughout the entire experiments have been computed and are depicted as the area under the curve (AUC).

### Thermal hypersensitivity – hargreaves Test

The thermal paw withdrawal latency (PWL) was used to assess hyperalgesia [29]. Briefly mice were acclimated for 20 min in separated transparent, bottom-free plastic chambers placed over the glass enclosure of the Hargreaves paw thermal stimulator (University of California, San Diego, CA). A heat source was positioned underneath the plantar surface of the hind paw (both ipsi- and contralaterally) and the time taken to withdraw from the heat source was automatically recorded by the apparatus. Each animal was stimulated 3 times with a 3 min inter-stimulation period. The PWL is averaged per animal and per paw. A cut-off was fixed at 20 s of stimulation to avoid any tissue damage. The experimenter responsible for conducting the behavioral tests was blind to the treatment, the genotype and the surgery performed on the mice. As mentioned before, PWL data have been computed as the AUC.

### Sulfasalazine administration

Animals were randomly assigned to either the treatment or control group. An 80 mg/mL stock solution of sulfasalazine (SAS, Sigma-Aldrich, St. Louis, MO) was prepared by dissolving the powder in DMSO. The final solution was daily prepared by adding saline to reach the desired concentration of SAS. The pH was adjusted using a small volume of NaOH 0.1 M. Mice were daily injected intraperitoneally (i. p.) with 200 mg/kg of SAS (or vehicle that includes the same dilution of DMSO for the controls) [30, 31] starting 2 days prior the sciatic nerve ligation and during the entire experiment until sacrifice, 3 or 7 days after surgery.

### Tissue collection

Mice were euthanized 3 or 7 days after sciatic nerve or sham surgery with CO_2_ and the spinal cord was immediately extruded with phosphate-buffered saline (PBS) as previously described [32]. The tissue from the lumbar enlargement of the spinal cord was isolated. A first longitudinal cut was performed along the ventral-median fissure exposing two halves corresponding to the ipsi- and contralateral sides. Another longitudinal cut along the central canal of each halves was performed to collect four quadrants. The dorsal quadrants were rapidly frozen in liquid nitrogen and finally stored at -80 °C before being processed for RNA extraction or synaptosome preparation. For immunohistochemistry, animals were transcardialy perfused with PBS before lumbar spinal cord collection.

### Total RNA extraction and real-time quantitative PCR (RT-qPCR)

Ipsi- and contralateral lumbar dorsal spinal cord samples were frozen and mechanically dissociated using a pre-chilled Teflon/glass homogenizer in TriPure isolation reagent (Roche, Bâle, Switzerland), followed by RNA extraction according to the manufacturer’s protocol. Reverse transcription was carried out with 1 µg of extracted RNA using the iScript cDNA synthesis kit (Bio-Rad Laboratories, Hercules, CA) in a total volume of 20 µL. Real-time qPCR amplifications were then performed using a 3-steps protocol with the Bio-Rad CFX Connect™ real-time PCR detection system (Bio-Rad Laboratories), in a total volume of 20 µL containing iTaq Universal SYBR® Green Supermix (Bio-Rad Laboratories), cDNA (equivalent to 10 ng retrotranscribed RNA) and a final concentration of 0.5 µM of each primer. Quantitative analysis was performed using the delta-delta Ct method, normalized to the relative expression of 3 different housekeeping genes (Ywhaz, RPL-19 and HPRT-1) previously validated for RT-qPCR studies on injured nervous tissues [33, 34]. The sequences of the primers used in this study are the following: for xCT, forward 5′- GATGCTGTGCTTGGTCTTGA − 3′ and reverse 5′- GCCTACCATGAGCAGCTTTC − 3′; for GFAP, forward 5’- GAGGGACAACTTTGCACAGG − 3’ and reverse 5’- TCCTCCAGCGATTCAACCTT − 3’; for Iba-1, forward 5’- GGATTTGCAGGGAGGAAAAG − 3’ and reverse 5’- TGGGATCATCGAGGAATTG − 3’; for NOX-2, forward 5’- TGAATGCCAGAGTCGGGATTT − 3’ and reverse 5’- CCCCCTTCAGGGTTCTTGATTT − 3’; for Arg-1, forward 5’- CGTGTACATTGGCTTGCGAG − 3’ and reverse 5’- ATCACCTTGCCAATCCCCAG − 3’; for RPL-19, forward 5’- TGACCTGGATGAGAAGGATGAG − 3’ and reverse 5’- CTGTGATACATATGGCGGTCAATC − 3’; for HPRT-1, forward 5’- CCTAAGATGAGCGCAAGTTGAA − 3’ and reverse 5’- CCACAGGACTAGAACACCTGCTAA − 3’; for Ywhaz, forward 5’- TAGGTCATCGTGGAGGGTCG − 3’ and reverse 5’- GAAGCATTGGGGATCAAGAACTT − 3’.

### Crude synaptosome preparation and [^3^H]-l-glutamate uptake assay

Frozen dorsal spinal cord samples were used for synaptosome preparation and for the measurement of system x_c_^−^ driven [^3^H]-l-glutamate transport. The method is based on the reversal uptake of glutamate when performed in the absence of extracellular cystine and in the absence of Na^+^, as validated in our previous study [35]. Briefly, the tissue was homogenized and centrifuged in ice-cold isotonic solution. The final pellet containing the synaptosomes was diluted in ice-cold buffer lacking Na^+^, in order to avoid any detection of EAAT-mediated glutamate uptake. Protein concentration was determined, and samples were diluted to achieve a final protein concentration of 33.33 µg/mL.

To assess the uptake, [^3^H]-l-glutamate (with specific activity of 48.6 Ci/mmol, Perkin Elmer, Waltham, MA) was used as substrate at a concentration of 20 nM. In a total volume of 500 µL, 10 µg of the synaptosome preparation was incubated with the substrate at 37 °C in a 96-well Masterblock (Greiner Bio-one, Kremsmünster, Austria). When indicated homocysteic acid (HCA, H9633, Sigma-Aldrich), a system x_c_^−^ inhibitor, was added to evaluate the HCA-dependent uptake. After 20 min incubation at 37 °C, the suspension was filtered (UniFilter GF/B, PerkinElmer), and washed with ice-cold Na^+^-free buffer. After drying overnight, a liquid scintillation solution, Microscint 20 (PerkinElmer) was added to each well and the radioactivity measurements were done using the Topcount® NXT Microplate scintillation and luminescence counter (PerkinElmer). Results are expressed as a percentage of the respective [^3^H]-l-glutamate uptake in the contralateral side.

### Immunohistochemistry

Lumbar spinal cords were used for immunohistochemistry as previously described by Gallo et al. with minor modifications [36]. Briefly, after tissue collection, lumbar spinal cords were fixed overnight in 4% paraformaldehyde in PBS. Cryoconservation was achieved by successively incubating the samples in 10%, 20% and 30% sucrose/PBS solution. Afterwards, spinal cords were frozen and stored at -80 °C until further use. For cryosectioning, samples were embedded in tissue Tek O.C.T (Sakura, Osaka, Japan) and transversal sections of 20 μm were cut using a CM3050S Leica cryostat (Leica Microsystems, Wetzlar, Germany). Cryosections were collected on Superfrost Plus object glass slides (Thermo Fisher Scientific, Waltham, MA) and stored at -20 °C until immunohistological staining. After a dry time of 1 h at room temperature (RT), sections were washed in PBS and incubated in a blocking buffer (5% normal goat serum, 0.5% Triton in PBS) for 1 h at RT. Sections were then incubated overnight at 4 °C with either of the following primary antibodies diluted in the blocking buffer: anti-Iba1 (rabbit, 1:1.000, Wako, Osaka, Japan, cat. no. 019/19,741 provided at 0.5 mg/mL) or anti-GFAP (chicken, 1:1.000, Abcam, Cambridge, UK, cat. no. ab4674 provided at 1 mg/mL). The next day, sections were rinsed in PBS, and were incubated for 2 h at RT with anti-rabbit or anti-chicken secondary antibodies conjugated to Alexa Fluor 647 (Thermo Fisher Scientific A21245 and A21449, respectively, both provided at 2 mg/ml and diluted 1:500 in PBS). Afterwards, sections were again washed with PBS and mounted on glass slides using EverBrite mounting medium (Biotium, Fremont, CA). Images were examined with an EVOS fluorescence microscope (AMG EVOS fl., Westburg, Leusden, Netherlands). The fluorescent intensity was quantified using the mean grey value of the defined area (ImageJ software).

### Statistical analyses

Data from biochemical studies (uptake assay, RT-qPCR, immunohistochemistry, and AUC), statistical analyses were performed on the difference in log-transformed ipsilateral versus contralateral measures for each mouse in order to control for individual variability. Normality was assessed using the Shapiro-Wilk normality test, and equality of variances was tested using the Levene test. Graphs depicting the back-transformed data are provided for all experiments to facilitate interpretation. As such, results are presented as geometric mean for biochemical endpoints and as arithmetic mean for behavioral endpoints. Statistical analyses were performed using one or two-way ANOVA model, depending on the experimental design. In case where a significant interaction was observed in the two-way ANOVA model, Tukey’s pairwise comparison method was employed. When appropriate and identified by the Grubb’s test, significant outliers were removed from the analysis set. For all analyses, a nominal two-sided Type I error of 0.05 was considered for statistical significance. Statistical analyses were conducted using GraphPad Prism 9.5.1 and JMP Pro 17 softwares.

## Results

### PSNL triggers increased xCT expression and system x_c_^-^ activity in the ipsilateral spinal cord

**Fig. 1:**
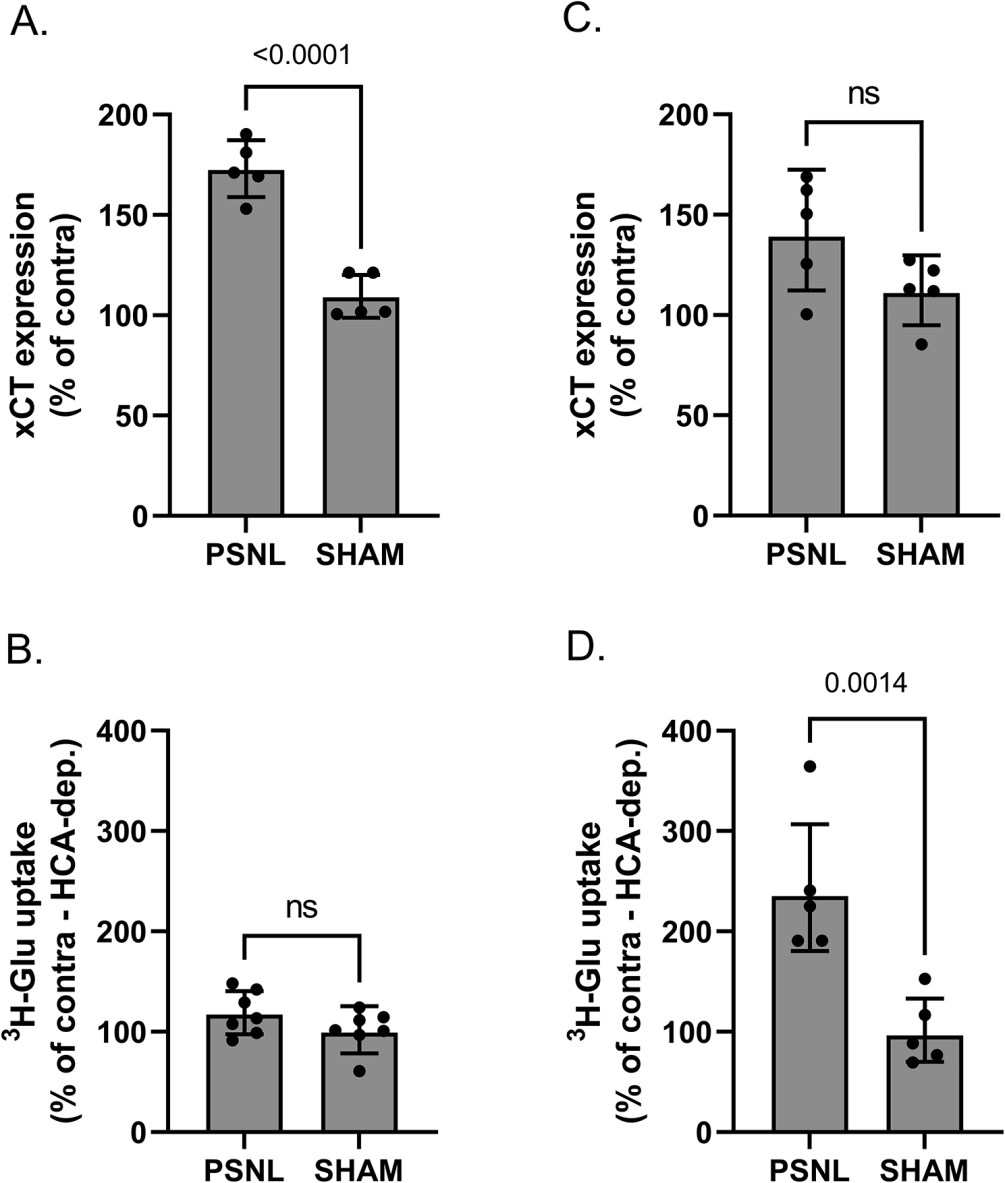
xCT expression and activity in the lumbar dorsal spinal cord following PSNL or sham surgery. Three (panel **A** & **B**) and seven (panel **C** & **D**) days post-surgery, the ipsilateral and contralateral dorsal horn quadrants of the lumbar spinal cord were dissected and used for RT-qPCR or functional assay. xCT mRNA was quantified, normalized to the mean of 3 housekeeping genes (RPL-19, Ywhaz, HPRT-1) and expressed as a percentage of the respective contralateral mRNA level (**A**, **C**). Uptake of ^3^H-L-glutamate on crude spinal synaptosomes was used to determine system x_c_^−^ functionality (**B**, **D**). Ipsilateral system x_c_^−^ specific uptake (HCA-dependent) is expressed as relative (%) to the respective contralateral side. As shown on the figure, data were obtained from 5 to 7 different animals per group and are presented as geometric mean with SD. Statistical analyses were conducted using a one-way ANOVA model. *PSNL* partial sciatic nerve ligation; *Glu* glutamate; *HCA* homocysteic acid

To examine the contribution of system x_c_^−^ in NP, the impact of a peripheral nerve lesion on the expression of the specific subunit xCT and system x_c_^−^ activity was assessed by RT-qPCR and ^3^H-glutamate uptake, respectively. Animals were sacrificed 3 days or 7 days after surgery and quadrant at the ipsi- and contralateral lumbar dorsal spinal cord were dissected and processed for analyses. Three days after PSNL, xCT mRNA level was significantly increased in the ipsilateral dorsal quadrant of the lumbar spinal cord. As shown in Fig. 1A and B, a 70% and 20% increase in lesioned mice as compared to the sham-operated mice was measured for xCT expression and system x_c_^−^ activity, respectively. This change in xCT expression appeared transient as it was absent from samples collected 7 days after surgery (Fig. 1C). However, the increased system x_c_^−^ activity was even more important in synaptosomes prepared from spinal cord samples 7 days after surgery (130% increase in the ipsilateral dorsal quadrant of lesioned mice as compared to the sham-operated mice) (Fig. 1D).

### Mice lacking xCT present a higher pain tolerance following PSNL

**Fig. 2:**
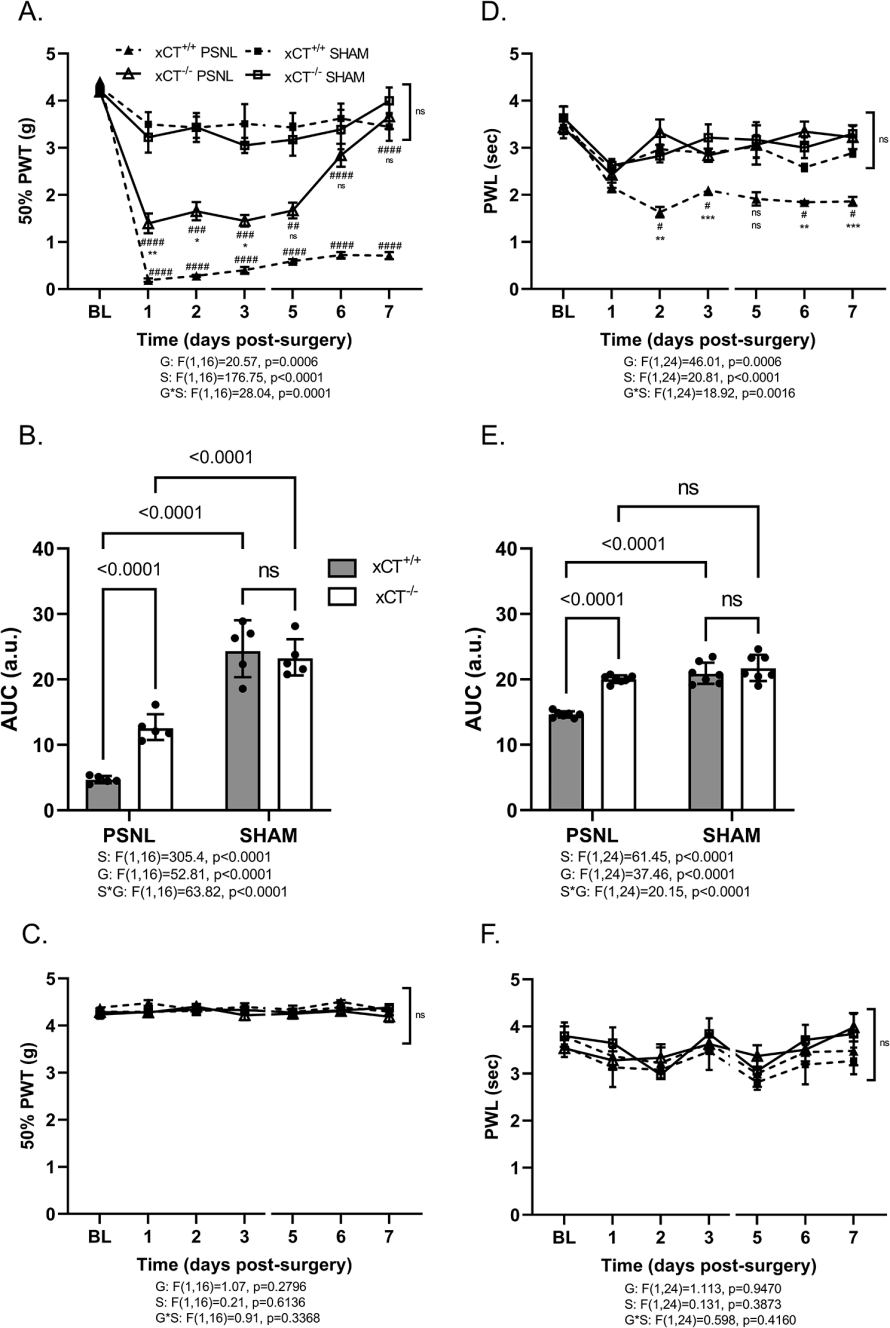
Allodynia and hyperalgesia in xCT^+/+^ and xCT^−/−^ mice following PSNL or sham surgery. Pain hypersensitivity was assessed upon stimulation of the ipsilateral (**A**, **B**, **D**, **E**) and contralateral (**C**, **F**) hind paw at baseline (BL) and for up to 7 days after surgery. The 50% PWT to mechanical stimulation was used to determine allodynia (**A**-**C**), whereas the PWL to thermal stimulation was used as a read-out of hyperalgesia (**D**-**F**). Panel **B** and **E** depict the area under the curve (AUC in arbitrary unit a.u.) of the ipsilateral 50% PWT and PWL, respectively. Data are presented as arithmetic mean with SEM (**A**, **C**, **D**, **F**) or geometric mean with SD (**B**, **E**) of 5 animals per group for allodynia and 7 animals per group for hyperalgesia. Statistical analyses were performed through a two-way ANOVA model (**B**, **E**) with repeated measures (**A**, **C**, **D**, **F**). Main and interaction effects are indicated below the graphs. When statistically significant interaction was detected, Tukey’s pairwise comparisons were conducted. P-values for significant effects are reported on the graphs (**B**, **E**). In panel **A** and **D**, * indicates a significant difference between lesioned wild-type (PSNL xCT^+/+^) and transgenic mice lacking xCT (PSNL xCT^−/−^), ^#^ indicate a significant difference between lesioned and non-lesioned animals (within the same genotype). *PSNL* partial sciatic nerve ligation; *PWT* paw withdrawal threshold; *PWL* paw withdrawal latency; *AUC* area under the curve; *S* surgery effect; *G* genotype effect; S*G interaction effect

The role of system x_c_^−^ in the development of pain sensitivity was examined by subjecting wild-type (xCT^+/+^) and xCT-deficient (xCT^−/−^) mice to unilateral PSNL. Female mice were monitored for 7 days post-injury using a von Frey (Fig. 2A-C) and a Hargreaves test (Fig. 2D-F) to respectively evaluate lesion-associated allodynia and hyperalgesia. In the absence of nerve lesion, mice lacking xCT did not present any signs of hypersensitivity to mechanical or thermal stimuli compared to their wild-type littermates. Also, sham-operated xCT^−/−^ mice showed similar PWT (Fig. 2A-C) and PWL (Fig. 2D-F) as compared to non-lesioned xCT^+/+^ mice. As shown in Fig. 2A, nerve-lesioned xCT^+/+^ animals exhibited prolonged and strong mechanical allodynia starting from day 1 after surgery. The 50% PWT reached the minimum value of 0.19 ± 0.04 g in injured xCT^+/+^ animals compared to 3.49 ± 0.26 g in the sham-operated animals. In mice lacking xCT, we also observed a decreased threshold following the sciatic nerve ligation (1.39 ± 0.21 g compared to 3.22 ± 0.32 g for sham-operated animals). However, this decrease was less pronounced compared to what was observed in xCT^+/+^ operated animals (Fig. 2A&B). Moreover, the PWT to mechanical stimulation returned to baseline values as from the sixth day after surgery, indicating that the hypersensitivity observed in xCT^−/−^ animals was transient. At variance, the decreased pain threshold observed in the xCT^+/+^ injured mice persisted for the entire duration of the experiment. No changes were observed in the contralateral paw when tested for mechanical sensitivity (Fig. 2C).

When testing the response to a thermal stimulus using the Hargreaves test, xCT^+/+^ mice undergoing PSNL developed a strong and persistent hyperalgesia as indicated by the decreased latency for ipsilateral paw withdrawal reaching 1.63 ± 0.11 s at 2 days post-injury (Fig. 2D). In contrast, mice lacking xCT did not develop any thermal hyperalgesia. Indeed, at all tested time points following the surgery, the PWL of injured xCT^−/−^ mice was similar to what was observed in sham-operated animals (AUC of 19.98 ± 0.39 for xCT^−/−^ injured mice and 21.75 ± 0.75 for uninjured xCT^−/−^ mice) (Fig. 2E). No changes were observed when testing the contralateral hind paw for thermal sensitivity (Fig. 2F).

### xCT^-/-^ mice show reduced astrogliosis and a distinct microglial polarization after PSNL

**Fig. 3:**
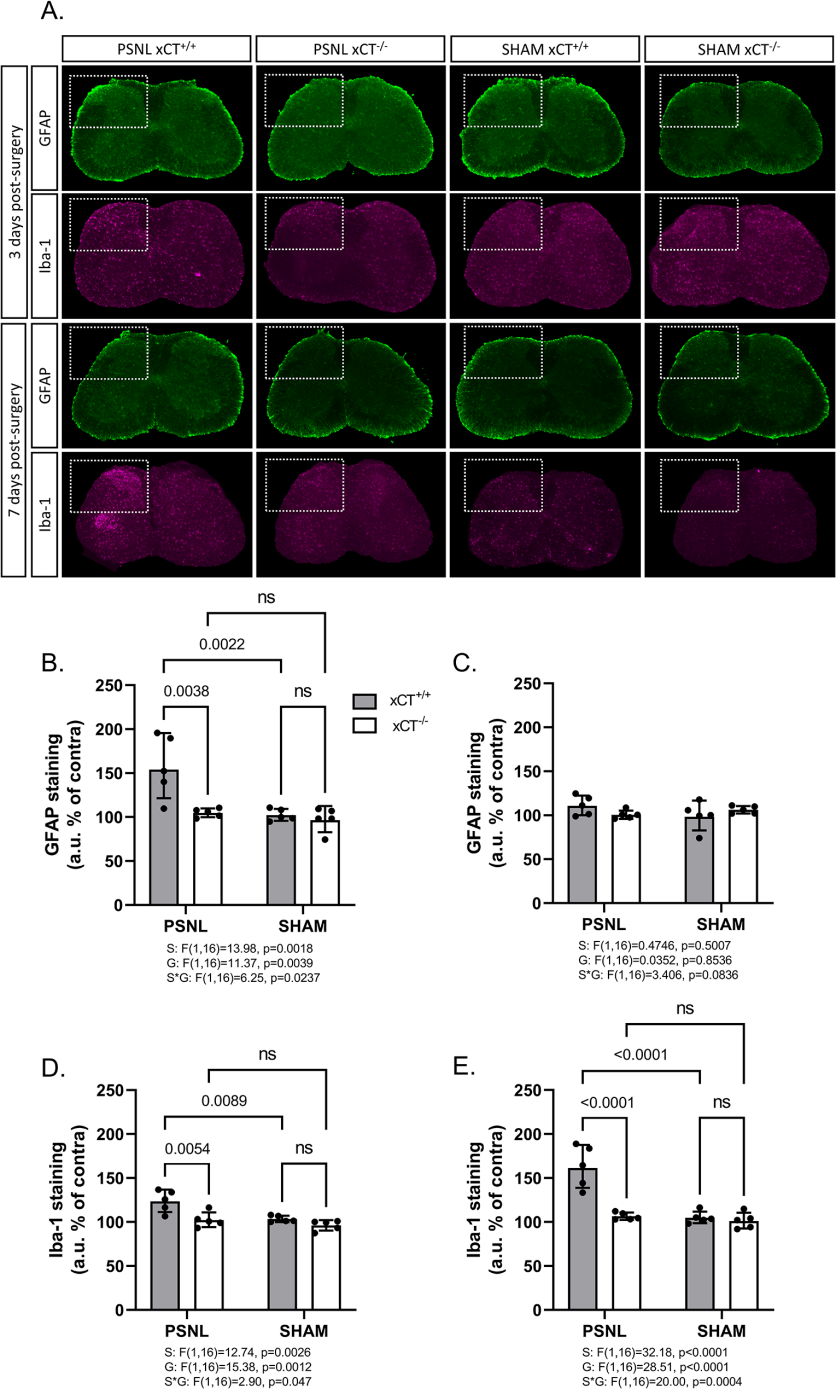
Glial activation in the spinal cord following PSNL or sham surgery in xCT^+/+^ and xCT^−/−^ mice. Immunohistological stainings for GFAP and Iba-1 were analyzed 3 (**A**, **B**, **D**) and 7 (**A**, **C**, **E**) days after the surgery in samples from wild-type or transgenic mice lacking xCT (**A**). The fluorescent intensity for GFAP (**B**, **C**) and Iba-1 (**D**, **E**) in the dorsal horns was quantified. The results from the ipsilateral side are normalized and expressed as a percentage of the respective contralateral side. Images shown are representative of 5 different animals and histograms represent the geometric means with SD. Statistical analyses were performed through a two-way ANOVA model. Main and interaction effects are indicated below the graphs. When statistically significant interaction was detected, Tukey’s pairwise comparisons were conducted. P-values for significant effects are reported on the graphs. *PSNL* partial sciatic nerve ligation; *GFAP* glial fibrillary acidic protein; *Iba-1* ionized calcium binding adapter protein 1; *S* surgery effect; *G* genotype effect; S*G interaction effect

**Fig. 4:**
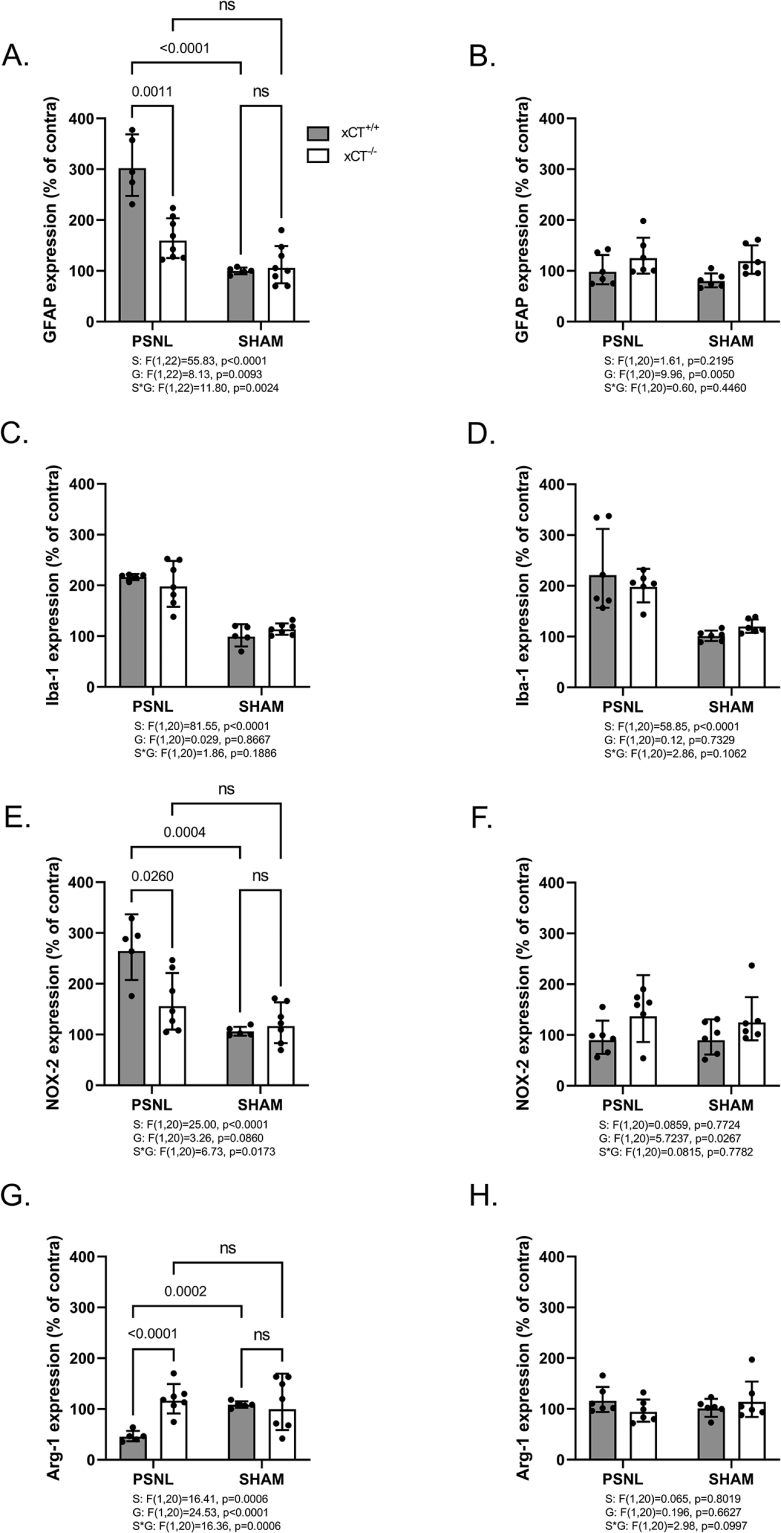
Assessment of spinal mRNA of glial markers following PSNL or sham surgery in xCT^+/+^ and xCT^−/−^ mice. Three (left panels) and seven (right panels) days post-surgery, the ipsilateral and contralateral quadrants of the dorsal horn of the lumbar spinal cord were dissected and used for RT-qPCR analyses. Specific markers were used to evaluate astrocytic (**A**, **B**) and microglial (**C**, **D**) activation, GFAP and Iba-1 respectively. Other markers were used to assess microglia activation profile towards pro-inflammatory (NOX-2: panel **E** & **F**) and anti-inflammatory (Arg-1: panel **G** & **H**) phenotype. For all markers, the mRNA was quantified, normalized to the mean of 3 housekeeping genes (RPL-19, Ywhaz, HPRT-1) and expressed as a percentage of the respective contralateral mRNA level. As shown on the figure, data were obtained from 5 to 7 different animals per group and are presented as geometric mean with SD. Statistical analyses were performed through a two-way ANOVA model. Main and interaction effects are indicated below the graphs. When statistically significant interaction was detected, Tukey’s pairwise comparisons were conducted. P-values for significant effects are reported on the graphs. *PSNL* partial sciatic nerve ligation; *GFAP* glial fibrillary acidic protein; *Iba-1* ionized calcium binding adapter protein 1; *NOX-2* NADPH oxidase 2; *Arg-1* Arginase 1; *S* surgery effect; *G* genotype effect; S*G interaction effect

Pain sensitization following peripheral nerve injury often coincides with pronounced glial activation in the ipsilateral spinal cord. Astroglial and microglial activations are commonly authenticated by evidencing an increased expression of the typical markers glial fibrillary acidic protein (GFAP) and ionized calcium-binding adapter molecule 1 (Iba-1), respectively. Changes in the protein expression (Fig. 3) and mRNA (Fig. 4) levels of both glial markers were therefore examined by immunohistochemistry on lumbar spinal cord sections and RT-qPCR on the dorsal quadrants of the lumbar spinal cord of xCT^+/+^ and xCT^−/−^ mice undergoing PSNL or sham surgeries. As expected, GFAP was significantly upregulated (1.5 fold-increase for protein level and up to 3 fold-increase for mRNA) in the spinal cord of xCT^+/+^ mice 3 days after unilateral surgery as compared to the contralateral side or to sham-operated animals (Fig. 3A, amp and B A). This was however not observed in samples collected 7 days after the surgery (Figs. 3C and 4B). The early upregulation of GFAP was less pronounced (1.6 fold-increase for mRNA) in xCT^−/−^ mice indicating a lower astrocytic activation following nerve surgery (Fig. 3B A).

Regarding microglial activation, a 2-fold increase in Iba-1 mRNA level was observed in the ipsilateral spinal cord of both xCT^+/+^ and xCT^−/−^ mice, 3- and 7-days post-surgery compared to the contralateral side or to the sham-operated animals (Fig. 4C&D). Iba-1 immunoreactivity was found to be increased (1.2-fold after 3 days and 1.6-fold after 7 days) in the ipsilateral spinal cord of xCT^+/+^ animals following PSNL compared to sham-operated animals or to the contralateral side. This, however, was not observed in transgenic animals lacking xCT where no alteration was detected (Fig. 3D&E).

Upon gliosis, microglial cells change their expression of several inflammation-related genes, and thereby adopt predominantly pro- or anti-inflammatory phenotypes [37]. The expression of NADPH oxidase 2 (NOX-2) and arginase 1 (Arg-1) was examined to monitor the polarization of reactive microglial cells in the spinal cord of mice after PSNL (Fig. 4E-H). In the ipsilateral dorsal quadrant of the lumbar spinal cord of xCT^+/+^ mice, 3 days after surgery, the expression of the pro-inflammatory marker NOX-2 was upregulated (2.8-fold increase) while the anti-inflammatory gene Arg-1 was downregulated (2-fold decrease). At variance, a trend for an upregulation of NOX-2 (1.5-fold increase) was detected in the ipsilateral spinal cord of xCT^−/−^ mice after PSNL, while no changes were observed regarding Arg-1 mRNA level (Fig. 4E&G). Additionally, in the lumbar spinal cord of xCT^+/+^ and xCT^−/−^ mice the expression of both inflammation-related microglial markers were unchanged when examined 7 days post-surgery (Fig. 4F&H).

### Sulfasalazine has an anti-allodynic and anti-hyperalgesic effect

**Fig. 5:**
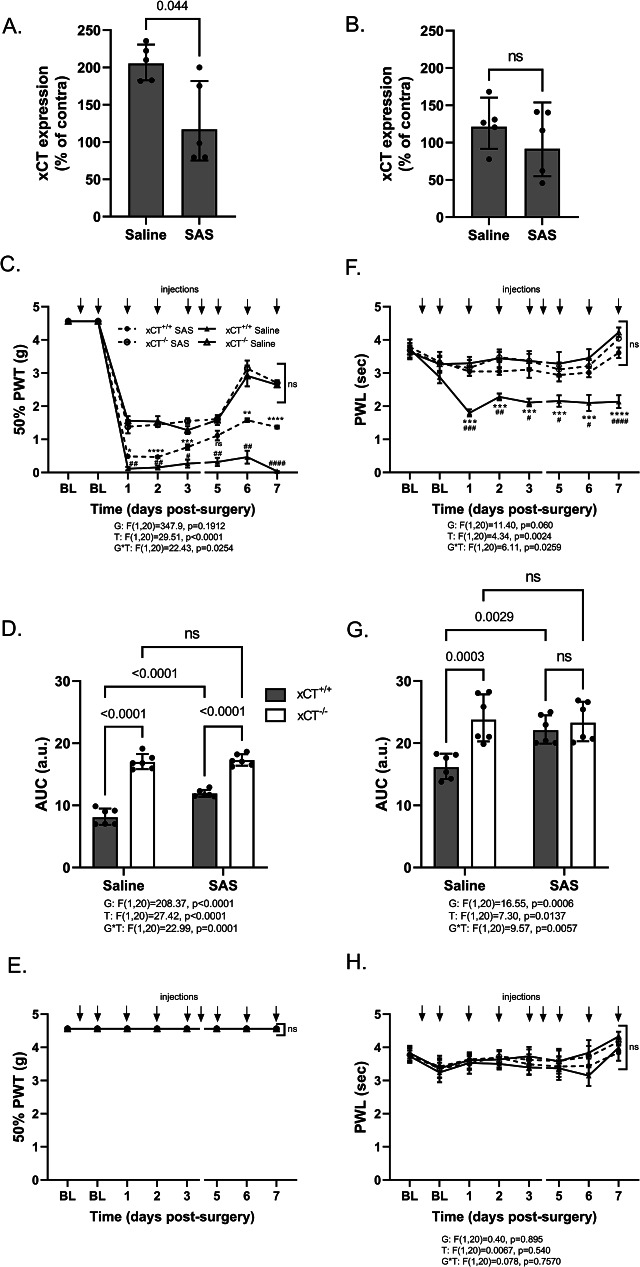
Effect of sulfasalazine administration on the spinal expression of xCT and the pain hypersensitivity following PSNL surgery. Three (**A**) and seven (**B**) days post-surgery, the ipsilateral and contralateral dorsal horn quadrants of the lumbar spinal cord of lesioned-xCT^+/+^ mice were dissected and used for RT-qPCR. xCT mRNA was evaluated, normalized to the mean of 3 housekeeping genes (RPL-19, Ywhaz, HPRT-1) and expressed as a percentage of the respective contralateral mRNA level. Pain hypersensitivity was assessed for the ipsilateral (**C**, **D**, **F**, **G**) and contralateral (**E**, **H**) hind paw at baseline (BL) and for 7 days following PSNL surgery. The 50% PWT to mechanical stimulation was used to determine allodynia (**C**-**E**), whereas the PWL to thermal stimulation was used as a read-out of hyperalgesia (**F**-**H**). Panel **D** and **G** depict the area under the curve for the ipsilateral 50% PWT and PWL, respectively. Data are presented as arithmetic mean with SEM (**C**, **E**, **F**, **H**) or geometric means with SD (**D**, **G**) (5 animal per group for mRNA analysis and 6 animal per group for behavioral tests). Statistical analyses were performed using a one-way ANOVA (**A**, **B**) or a two-way ANOVA (**D**, **G**) model with repeated measures (**C**, **E**, **F**, **H**). Main and interaction effects of the two-way ANOVA model are indicated below the graphs. When statistically significant interaction was detected, Tukey’s pairwise comparisons were conducted. P-values for significant effects are reported on the graphs. In panel **C** and **F**, * indicates a significant difference between SAS-treated wild-type (xCT^+/+^ SAS) and SAS-treated transgenic mice lacking xCT (xCT^−/−^ SAS), ^#^ indicate a significant difference between SAS-treated and saline-treated animals (within the same genotype). *PSNL* partial sciatic nerve ligation; *PWT* paw withdrawal threshold; *PWL* paw withdrawal latency; *AUC* area under the curve; *I* ipsilateral; *C* contralateral; *SAS* sulfasalazine; *G* genotype effect; *T* treatment effect; G*T interaction effect

To assess the potential of targeting system x_c_^−^ in preventing the development of NP, a pharmacological approach was employed. Both xCT^+/+^ and xCT^−/−^ mice subjected to PSNL surgery were treated daily with the inhibitor of system x_c_^−^, SAS, or with vehicle. Treatment was initiated 2 days before PSNL and pursued until sacrifice. Mice were monitored daily for allodynia and hyperalgesia for up to 3 or 7 days after surgery, at which stage spinal cord tissue was collected for RT-qPCR analysis. As mentioned before, 3 days after PSNL, the mRNA level of xCT was significantly increased in the ipsilateral dorsal quadrant of the lumbar spinal cord (2-fold increase as compared to the contralateral quadrant). This change in xCT expression was nearly completely reversed by the administration of SAS (Fig. 5A). Also, this effect appeared transient as xCT mRNA level remained unchanged 7 days post-surgery and SAS treatment was without influence at that stage (Fig. 5B).

Regarding the influence of the treatment on the pain-associated behaviors, it was observed that baseline measurements of mechanical and thermal pain thresholds remained unaltered by SAS treatment in the absence of surgery (Fig. 5C-H). Consistent with here-above detailed data, vehicle-treated xCT^+/+^ mice undergoing PSNL presented a robust ipsilateral mechanical allodynia (Fig. 5C-E) and thermal hyperalgesia (Fig. 5F-G) that persisted for at least 7 days. Conversely, xCT^+/+^ mice receiving SAS showed a reduced hypersensitivity to the mechanical stimulus as they presented a higher 50% PWT compared to vehicle-treated mice (AUC of 11.93 ± 0.23 for SAS-treated mice and 8.18 ± 0.53 for control group, *p* < 0.0001) (Fig. 5C&D). No changes were observed when testing the contralateral paw for mechanical sensitivity (Fig. 5E). Besides, when challenged with thermal stimulation, SAS-treated xCT^+/+^ mice did not develop any signs of thermal hypersensitivity. Indeed, at all tested time points, the PWL was similar to the values observed at baseline (Fig. 5F-H). To confirm that the observed effects were specifically driven by the inhibition of system x_c_^−^, lesioned-mice lacking xCT were also subjected to the same SAS treatment. In both behavioral tests, no significant differences in the pain thresholds were observed when comparing SAS- and vehicle-treated xCT^−/−^ mice.

### Sulfasalazine reduces astrocytic and microglial activation after PSNL

**Fig. 6:**
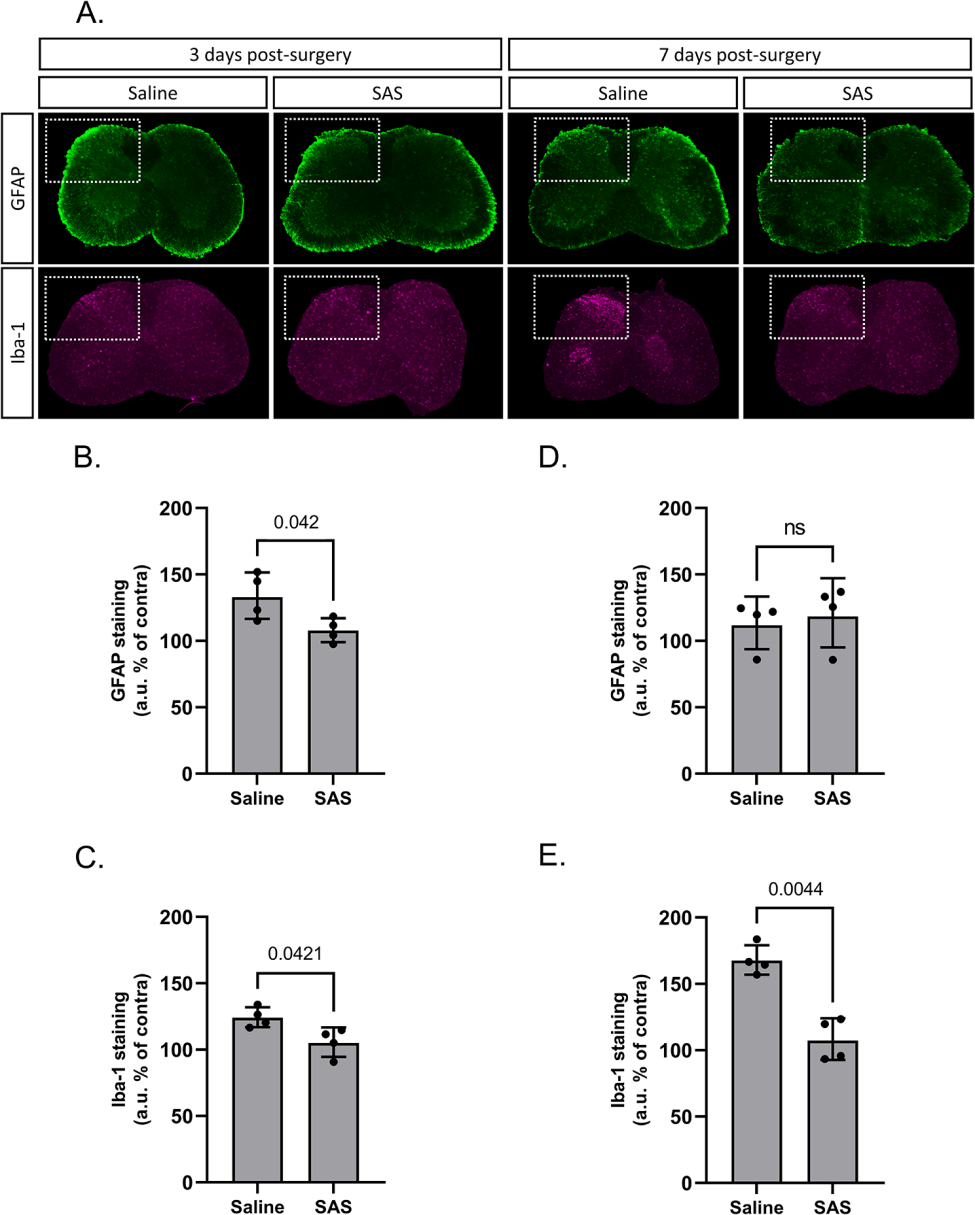
Effect of sulfasalazine administration on the spinal glial reactivity following PSNL surgery. Immunohistological staining of GFAP and Iba-1 was performed on lumbar spinal cord samples of xCT^+/+^ mice at 3 (**A**, **B**, **C**) and 7 days (**A**, **D**, **E**) after surgery. The fluorescent intensity for GFAP (**B**, **D**) and Iba-1 (**C**, **E**) in the dorsal horns was quantified. The results from the ipsilateral side are normalized and expressed as a percentage of the respective contralateral side. Images shown are representative of 4 different animals per group and histograms represent the geometric means with SD. Statistical analyses were performed using a one-way ANOVA model. *GFAP* glial fibrillary acidic protein; *Iba-1* ionized calcium binding adapter protein 1; *SAS* sulfasalazine

**Fig. 7:**
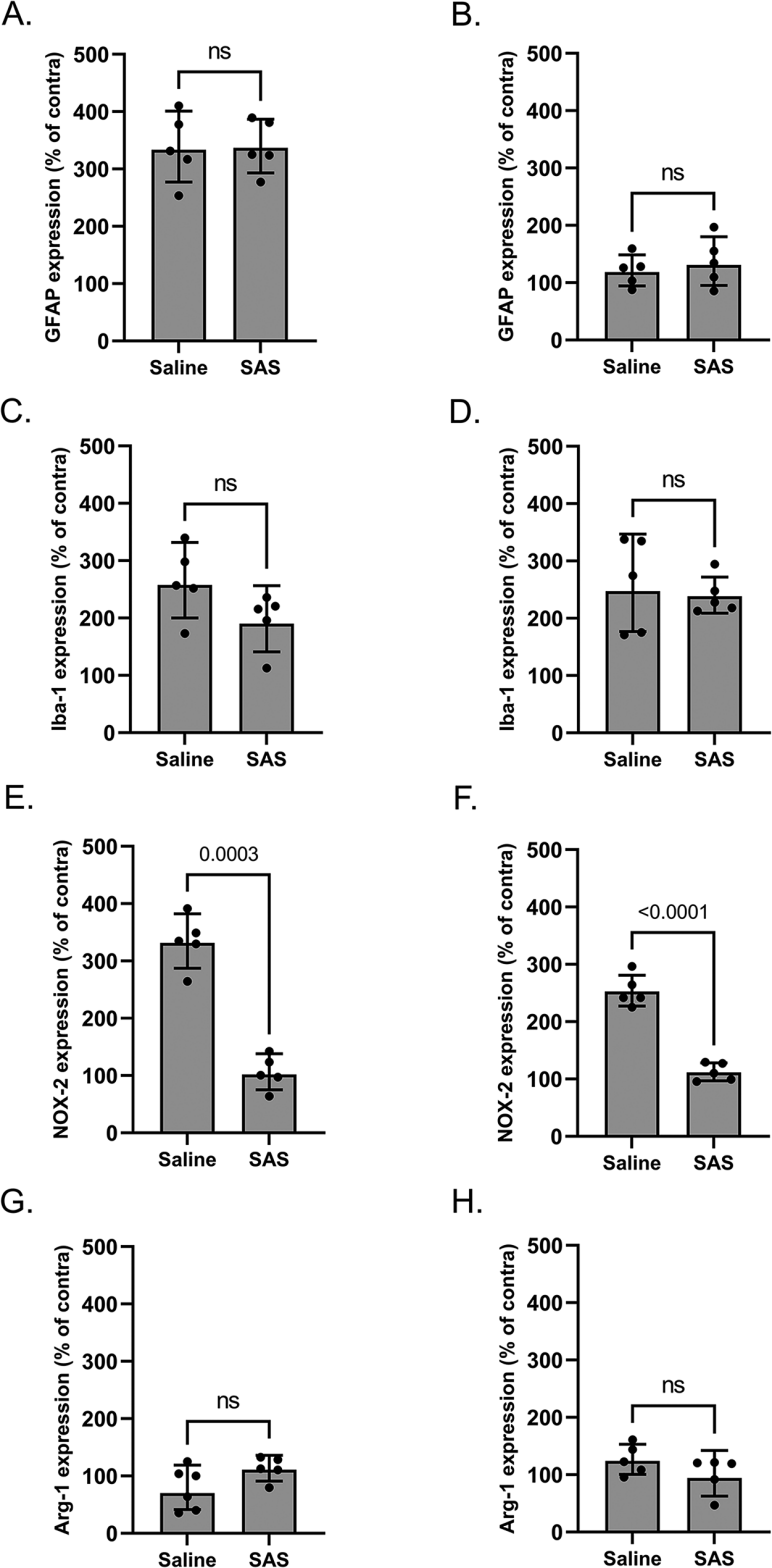
Effect of sulfasalazine on spinal mRNA of astrocytic and microglial markers after PSNL surgery in xCT^+/+^ mice. Three (left panels) and seven (right panels) days post-surgery, the ipsilateral and contralateral quadrants of the lumbar spinal cord were dissected and used for RT-qPCR analysis. GFAP and Iba-1 were used to evaluate astrocytic (**A**, **B**) and microglial (**C**, **D**) activation, respectively. Other markers were used to assess microglia activation profile towards pro-inflammatory (NOX-2: panel **E** & **F**) and anti-inflammatory (Arg-1: panel **G** & **H**) phenotype. For all markers, the mRNA levels were normalized to the mean of 3 housekeeping genes (RPL-19, Ywhaz, HPRT-1) and expressed as a percentage of the respective contralateral mRNA level. Data were obtained from 5 different animals per group and are presented as geometric means with SD. Statistical analyses were performed using a one-way ANOVA model. *GFAP* glial fibrillary acidic protein; *Iba-1* ionized calcium binding adapter protein 1; *NOX-2* NADPH oxidase 2; *Arg-1* Arginase 1; *SAS* sulfasalazine

The effects of SAS treatment on the PSNL-induced glial activation were examined by immunohistochemistry (Fig. 6) and RT-qPCR (Fig. 7) for GFAP and Iba-1, in the dorsal quadrants of the lumbar spinal cord of xCT^+/+^ mice daily treated with either SAS or vehicle. As previously described, lesioned-mice displayed increased ipsilateral expression of GFAP and Iba-1 (Fig. 6A). Intraperitoneal treatment with SAS strongly reduced immunoreactivity of both glial markers (Fig. 6B-E) particularly with respect to Iba-1 staining, 7 days after the surgery (Fig. 6E). These changes were not observed by RT-qPCR (Fig. 7A-D). However, pharmacological inhibition of system x_c_^−^ leads to a significant reduction of NOX-2 expression (from 333.19 ± 51.54% for the vehicle-treated mice to 109.86 ± 40.76% for the SAS-treated group 3 days after the surgery, *p* < 0.01) (Fig. 7E&F). No significant difference was observed concerning the expression of Arg-1, the marker used to monitor the anti-inflammatory phenotype of microglia (Fig. 7G&H).

## Discussion

Peripheral nerve injuries often trigger maladaptive plasticity in nociceptive pathways, resulting in the pathological amplification of excitatory transmission. This is well documented in the dorsal horn of the spinal cord, which serves as the first relay center for integrating and regulating nociceptive information, contributing to the development of chronic pain [5, 38]. The reinforcement of glutamate transmission can be attributed to different mechanisms, such as an enhanced neuronal release of glutamate causing an increased activation of glutamate receptors and associated downstream signaling pathways [39, 40]. Besides, impaired glutamate clearance by high affinity glial transporters is also documented [9, 10]. Modulating the molecular mechanisms that regulate glutamate transmission holds promise in preventing or alleviating chronic pain. Indeed, drugs that enhance glutamate transporter activity or antagonizing glutamate receptors were proven effective in models of NP, but potential side effects would restrict their clinical use [41–43].

In addition to extensive research on glutamate transporters, accumulating evidence has recently emphasized the role of system x_c_^−^ in modulating glutamate transmission [44]. Widely distributed throughout the CNS, this cystine-glutamate exchanger responsible for the non-vesicular glutamate release is not only implicated in the modulation of excitatory signals, but also in the defense against oxidative stress and neuroinflammation [45, 46]. This dual role played by system x_c_^−^ supports a putative involvement in diverse neurological pathologies, where both dysregulation of glutamate signaling and persistent neuroinflammation contribute to neuronal damages [15]. This was also evidenced in a model of bone cancer pain where increased expression of system x_c_^−^ supports the release of glutamate, contributing to the activation of sensory neurons [47]. In this context, the present study using a model of peripheral nerve lesion provides compelling evidence on the role played by this exchanger in the physio-pathological mechanisms of NP.

In both rats and mice, the PSNL is a commonly used model of surgical nerve lesion to study NP. Within a few hours after the nerve ligation, rodents exhibit signs of thermal hyperalgesia, mechanical allodynia and spontaneous pain that persist for several months [48]. This lesion has been reported to trigger plasticity in the primary afferent fibers and sustained neuroinflammation in the dorsal lumbar spinal cord, a hallmark of central sensitization [10]. In the present study, the invalidation of system x_c_^−^ appeared to influence both the amplitude and the duration of the pain sensitization after PSNL. Compared to wild-type mice, xCT knock-out mice exhibited reduced sensitivity to mechanical stimulation from the first day post-surgery, with pain sensitivity returning to baseline within just 6 days.

While the nociceptive behavior of mice lacking xCT was improved at both early and late stages after ligation, it is noteworthy that the upregulation of xCT mRNA in the spinal cord of wild-type animals was primarily observed at early stage after PSNL. This suggests a predominant implication of system x_c_^−^ at the onset of the sensitization process. Hence, in the model of bone cancer-induced pain, the inhibition of system x_c_^−^ was shown to delay the onset of nociception [47]. Accumulating evidence indicates that an inadequate management of acute pain can lead to its chronicisation and that early changes in the pain pathways can lead to later plastic changes responsible for persistent pain [49]. This putative implication of xCT at the onset of NP is consistent with the observation that knock-out mice do not develop any sign of thermal hyperalgesia following PSNL. Indeed, if xCT^+/+^ and xCT^−/−^ mice had initially exhibited similar hypersensitivity within a few hours but showed a faster resolution in mice lacking xCT, it would suggest a later involvement of system x_c_^−^. While further experiments are needed to ascertain the specific role of system x_c_^−^ in the different stages of NP. Our observations hold significant implication regarding the opportunity to target system x_c_^−^ in the management or prevention of NP.

The beneficial effect of the genetic suppression of xCT on the development and persistence of allodynia and hyperalgesia in the surgical model of NP is consistent with the observation that such lesion triggers an increased expression of xCT in the dorsal spinal cord. Nevertheless, the use of a constitutive transgenic mice strain in which xCT expression is absent during the entire animal development raises questions related to compensatory mechanisms [50]. To validate the findings from xCT-deficient animals, the pharmacological inhibition of system x_c_^−^ in adult mice constitutes a relevant alternative approach. Indeed, considering the upregulation of xCT and the enhanced system x_c_^−^ activity following sciatic nerve ligation, targeting this exchanger might help to reduce NP symptoms. Nowadays, the most used inhibitor of system x_c_^−^ is SAS, an EMA- and FDA-approved drug prescribed to treat inflammatory diseases of the intestine [51, 52]. Effectiveness in these disorders arises from its breakdown by the gut microbiota, generating anti-inflammatory metabolites. As only the intact parent molecule was proven effective in inhibiting system x_c_^−^ [52–54], SAS was herein administrated intraperitoneally to reduce its metabolization. Even though some papers reported on a limited capacity of SAS to cross the blood-brain barrier, several researches have demonstrated beneficial effects in nervous disorders affecting both the brain and the spinal cord such as reduced microglial activation and reduced tumor growth in mouse model of glioblastoma [53, 55, 56]. Given that system x_c_^−^ putatively participates in the onset of NP, SAS was administered preventively, starting 2 days before the lesion. In these conditions, a robust anti-allodynic and anti-hyperalgesic effect of SAS was observed in xCT^+/+^ mice undergoing PSNL. Even though these data are consistent with the results obtained with knock-out animals, pointing out xCT as a key actor in the pain sensitization, a possible anti-inflammatory component of the treatment cannot be ruled out. Thus, some evidence indicated that SAS itself possess anti-inflammatory properties through an inhibition of the transcription factor NF-κB involved in immune responses [57, 58]. Surprisingly, SAS, being a system x_c_^−^ inhibitor, led to a downregulation of xCT mRNA. This post-translational regulation might be attributed to its anti-inflammatory properties, resulting in reduced recruitment of astrocytes and microglia which are known to upregulate xCT during inflammatory conditions. To confirm the specific role of xCT, SAS was administered to xCT^−/−^ mice, but no analgesic effect was observed compared to the vehicle-treated xCT^−/−^ mice. Together, these data underscore the relevance of targeting system x_c_^−^ in NP but call for the development of more specific inhibitors to mitigate potential side effects as previously reported with SAS [30].

Knowing that system x_c_^−^ is mainly expressed in glial cells and that over the past decades, both peripheral and central glia and immune cells have taken center stage in research on NP, the specific implication of astrocytes and microglial cells was examined. The upregulation of xCT following PSNL surgery, was correlated with the activation of astrocytes and microglia in the dorsal spinal cord. Resident glial cells actively participate in the strong inflammatory reaction in models of NP by releasing mediators such as TNFα, interleukins, chemokines, ATP. This neuroinflammatory cascade is further propagated by the recruitment of microglial cells and astrocytes [59]. After both peripheral or central nerve lesions, these cells promote neuroinflammation at several levels of the pain pathway leading to the onset and progression of pain hypersensitivity. While Iba-1 is primarily recognized for its role in the initiation of NP and GFAP for its maintenance [3, 60], results are not always straightforward as temporal dynamic changes may differ across pain models or spinal regions [61]. At variance with several published data, our findings unveil a rapid and transient upregulation of GFAP early after the nerve lesion, prompting further investigation into the underlying chronology of this glial response. In line with the existing literature, our experimental findings regarding the persistent upregulation of Iba-1 imply that microglial activation may persist for an extended period, potentially even beyond pain resolution [62]. Emerging evidence suggests that specific subtypes of immune cells can reduce pain and contribute to the resolution of NP [48, 63]. Previous studies have evidenced that activated microglia show increased system x_c_^−^ expression and that the genetic invalidation of xCT alters their polarization profile, predominantly adopting a protective anti-inflammatory (M2) phenotype in the spinal cord of animal models of neurological diseases such as amyotrophic lateral sclerosis [19] or spinal cord injury [64]. In line with this observation, our findings indicate that mice lacking xCT exhibit a different polarization profile of microglia as compared to their wild-type counterparts, suggesting that the absence of system x_c_^−^ could beneficially balance the strong pro-inflammatory activation state commonly observed in the context of NP. Accordingly, an altered recruitment and activation of glial cells in the spinal cord following nerve lesion was observed after pharmacological inhibition of system x_c_^−^ with SAS. Together with a reduced astrogliosis and microgliosis, a reduced polarization toward the pro-inflammatory microglial phenotype was observed. This evidence aligns with the results of behavioral testing indicating reduced pain sensitivity with SAS treatment as compared to vehicle-treated animals.

From an experimental standpoint, our observations highlight the need for complementary studies that would reinforce the therapeutic potential offered by blockers of system x_c_^−^. With a focus on the implication of system x_c_^−^ during the onset of NP, the present study examined animal behaviour and biochemical changes during the first week after the surgical nerve lesion. Previous studies have shown that peripheral nerve lesions generate long-lasting sensitization [65] and the implication of system x_c_^−^ in the persisting behaviour and associated biochemical changes should also be examined at later time points. Further studies could therefore consider initiating the administration SAS after the establishment of NP to assess the role of system x_c_^−^ in the pain maintenance. This also underscores the opportunity of employing conditional genetic models where xCT can be silenced at later time points. Besides, it is worth mentioning that the present findings were obtained using female mice only, in order to reduce experimental variability. Hence, pain hypersensitivity in models of chronic pain, including from a neuropathic origin is sex-dependent and further studies should include both males and females [66, 67]. Finally, previous work has revealed that xCT-deficient mice show reduced anxiety and depressive-like behaviours, supporting a role for this exchanger in several centrally controlled behaviours [68]. It is noteworthy that anxiety and depression are comorbidities of chronic pain and one could therefore anticipate a possible impact of inactivating or inhibiting system x_c_^−^ [69] on brain-controlled behaviours associated with the neuropathic lesion. In a broader perspective, further studies should examine whether manipulating system x_c_^−^ may influence the processing of pain in supra-spinal centers.

## Conclusion

Altogether, these findings provide strong evidence for the important contribution of system x_c_^−^ in the establishment of a persistent pathological pain state and spinal neuroinflammation after peripheral nerve lesion. This not only helps to better understand the molecular mechanisms supporting NP but offers new perspectives for future therapies of this major clinical issue. Targeting system x_c_^−^ appears as a unique opportunity to tackle both the altered glutamate transmission and the neuroinflammation responsible for central sensitization in patients suffering from NP. The pharmacological manipulation of system x_c_^−^ could therefore prevent, reduce, or alleviate NP symptoms such as allodynia and hyperalgesia and thereby improve the quality of life of affected patients.

## Data Availability

All data generated and analyzed during this study are included in this published article.

## Abbreviations

Arg-1: arginase 1
AUC: area under the curve
CNS: central nervous system
EAATs: high-affinity glutamate transporters
GFAP: glial fibrillary acidic protein
HCA: homocysteic acid
Iba-1: ionized calcium-binding adapter molecule 1
NOX-2: NADPH oxidase 2
NP: neuropathic pain
PBS: phosphate-buffered saline
PSNL: partial sciatic nerve ligation
PWL: paw withdrawal latency
PWT: paw withdrawal threshold
RT: room temperature
RT-qPCR: real-time quantitative polymerase chain reaction
SAS: sulfasalazine
SEM: standard error of the mean
